# The *Interleukin-10* Promoter Polymorphism rs1800872 (-592C>A), Contributes to Cancer Susceptibility: Meta-Analysis of 16 785 Cases and 19 713 Controls

**DOI:** 10.1371/journal.pone.0057246

**Published:** 2013-02-27

**Authors:** Qi Ding, Ying Shi, Bo Fan, Zhijiang Fan, Li Ding, Feng Li, Wenjian Tu, Xiaohua Jin, Jing Wang

**Affiliations:** 1 Department of Urology, The Changshu Hospital Affiliated to Soochow University, Changshu, China; 2 Department of Gastroenterology, The Changshu Hospital Affiliated to Soochow University, Changshu, China; San Francisco Coordinating Center, United States of America

## Abstract

Interleukin-10 (IL-10) is a multifunctional cytokine which participates in the development and progression of various malignant tumors. To date, a number of case–control studies were conducted to detect the association between *IL-10*-592C>A polymorphism and cancer risk in humans. However, the results of these studies on the association remain conflicting. In an effort to solve this controversy, we performed a meta-analysis based on 70 case–control studies from 65 articles, including 16 785 cancer cases and 19 713 controls. We used odds ratios (ORs) with 95% confidence intervals (CIs) to assess the strength of the association. The overall results suggested that the variant homozygote genotype AA of the *IL-10*-592C>A polymorphism was associated with a moderately decreased risk of all cancer types (OR = 0.90, 95% CI = 0.83–0.98 for homozygote comparison, OR = 0.92, 95% CI = 0.86–0.98 for recessive model). In the stratified analyses, the risk remained for studies of smoking-related cancer, Asian populations and hospital-based studies. These results suggested that the *IL-10*-592C>A polymorphism might contribute to the cancer susceptibility, especially in smoking-related cancer, Asians and hospital-based studies. Further studies are needed to confirm the relationship.

## Introduction

Cancer is a major public health problem in the world. Evidences support an important role for genetics in determining risk for cancer. Association studies are appropriate for searching susceptibility genes involved in cancer [1].

Interleukin-10 (IL-10) is a multifunctional cytokine which participate in the development and progression of various malignant tumors [2]. It has anti-inflammatory and immunosuppressive activities including the ability to downregulate the expression of macrophage costimulatory molecule. The impact of IL-10 on macrophage function appears to play a role in the growth of blood vessel, as studies showed that IL-10 might contribute to the regulation of angiogenesis in many kinds of tumors [3], [4]. As an immunosuppressive molecule allowing tumor to escape from immune surveillance, IL-10 might act as a potential tumor promoter which results in a more aggressive behavior of malignant cells. Conversely, due to its immune-stimulating and anti-angiogenic properties, IL-10 is supposed to prevent or reduce the growth and distant spread of tumor [5]–[7].It has been indicated that IL-10 overexpression as well as deficiency was found under different pathophysiological conditions depending on the cancers analyzed [8].

IL-10 is encoded by a gene located on chromosome 1 (1q31–1q32) [9]. The *IL-10* promoter is highly polymorphic and three important single nucleotide polymorphisms (SNPs) in the promoter region which influence the transcription of *IL-10* messenger RNA and the expression of IL-10 in vitro are rs1800896(-1082A>G), rs1800871 (-819C>T), and rs1800872(-592C>A) [10], [11]. It has been reported that the -1082A>G and haplotype (-1082_-819_-592) were associated with differential production of the protein in stimulated cells, with the ATA haplotype leading to decreased IL-10 expression and the GCC haplotype increased IL-10 expression [12].

To date, a number of case-control studies were conducted to investigate the association between *IL-10* -592 C>A and cancer risk in humans [13]–[77]. However, the results of these studies remain conflicting rather than conclusive. So, we performed the present meta-analysis to evaluate the association between *IL-10* -592 C>A and cancer risk.

## Materials and Methods

### Identification and Eligibility of Relevant Studies

We searched the electronic literature from Pubmed for all relevant reports (the last search update was Oct 16, 2012), using the key words: (“interleukin-10” or “IL-10” or “IL10”) and (“variant” or “variation” or “polymorphism”) and (“cancer” or “tumor” or “carcinoma” or “malignancy”). The search was limited to English language papers. In addition, studies were identified by a manual search of the reference lists of reviews and retrieved studies. Studies were selected if there was available data for the *IL-10* -592C>A polymorphism with cancer risk in a case-control design including retrospective or prospective and nested case-control studies. As studies with the same population by different investigators or overlapping data by the same authors were found, the most recent or complete articles with the largest numbers of subjects were included. Studies included in our meta-analysis have to meet the following criteria: (i) evaluation of the *IL-10* -592C>A polymorphism and cancer risk, (ii ) use a case-control design (retrospective or prospective and nested case-control) and (iii ) contain available genotype frequency. Major reasons for exclusion of studies were (i ) only case population and (ii ) duplicate of previous publication.

### Data Extraction

Two of the authors extracted all data independently complying with the selection criteria and reached a consensus on all items. In the present study, the following characteristics were collected: the first author’s last name, year of publication, country of origin, ethnicity, frequencies of genotyped in cases and controls, source of control groups (population- or hospital-based controls) and cancer type. For studies including subjects of different ethnic groups, data were extracted separately for each ethnic group whenever possible [43]. Different ethnic descents were categorized as European, Asian, or African or mixed (composed of an admixture of different ethnic groups). Meanwhile, studies investigating more than one kind of cancer were counted as individual data sets only in subgroup analyses by cancer type [15], [36], [39], [59]. One study which was duplicate of previous publication were excluded from the analysis [78].

### Statistical Analysis

The strength of the association between the *IL-10* -592C>A polymorphism and cancer risk was measured by odds ratios (ORs) with 95% confidence intervals (CIs). The statistical significance of the pooled OR was determined using the Z-test. Pooled estimates of the OR were obtained by calculating a weighted average of OR from each study [79]. First, we estimated cancer risks with the CA and AA genotypes, compared with the wild-type CC homozygote, and then evaluated the risks associated with CA/AA versus CC and AA versus CC/CA, assuming the dominant and recessive effects of the variant A allele, respectively. Stratified analyses were also performed by cancer types (if one cancer type contained less than three individual studies, it was combined into ‘other cancers’ group), ethnicity and source of controls. In consideration of the possibility of heterogeneity across the studies, *I* ^2^ was applied to assess heterogeneity between studies [80]. Values from single study were combined using models of both fixed effects and random effects [81]. We used a fixed effects model when *I* ^2^ was equal or less than 50%, and a random effects model when *I* ^2^ was greater than 50%. In the absence of heterogeneity, the two methods provide identical results, because the fixed effects model, using the Mantel–Haenszel’s method, assumes that studies are sampled from populations with the same effect size, making an adjustment to the study weights according to the in-study variance; whereas the random-effects model using the DerSimonian and Laird’s method assumes that studies are taken from populations with varying effect sizes, calculating the study weights both from in-study and between-study variances, considering the extent of variation or heterogeneity. Sensitivity analyses were performed to assess the stability of the results, namely, a single study in the meta-analysis was deleted each time to reflect the influence of the individual data set to the pooled OR. Funnel plots and Egger’s linear regression test were used to provide diagnosis of the potential publication bias [82]. All analyses were conducted using Stata software (version8.0; StataCorp LP, College Station, TX), and all tests were two sided.

## Results

### Characteristics of Studies

**Figure 1:**
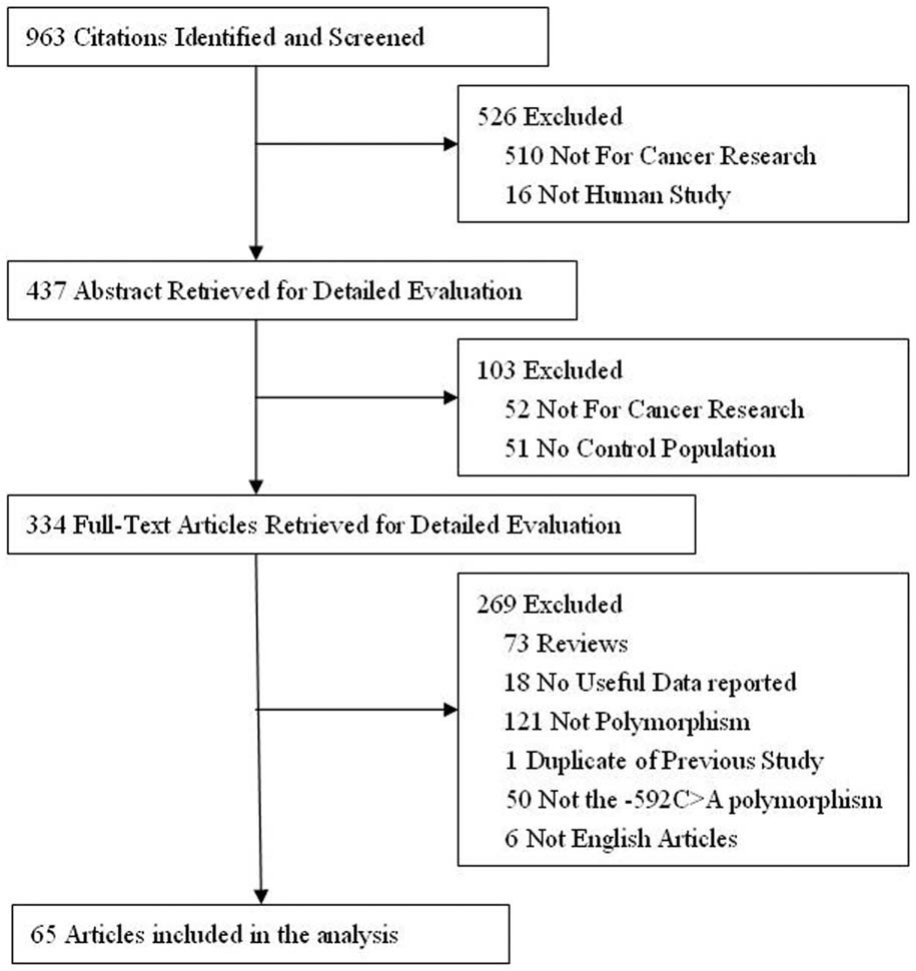
Studies identified with criteria for inclusion and exclusion.

Finally, a total of 70 studies from 65 articles that included a total of 16 785 cancer cases and 19 713 controls met the inclusion criteria (Fig. 1). Study characteristics are summarized in Table 1. For the *IL-10*-592C>A polymorphism, there were 2 studies of African descendents, 20 studies of Asian descendents and 37 studies of European descendents. In our meta-analysis, most of the cancer types were gastric cancer. Cancers were confirmed histologically or pathologically in most studies. The distribution of genotype in the controls of the studies was in agreement with Hardy-Weinberg equilibrium for all except five studies [14], [20], [21], [27], [72], which were further tested in the sensitivity analyses.

**Table 1:** Characteristics of studies included in the meta-analysis.

First author	Year	Cancer type	Country	Ethnicity	Source of control groups	Cases/controls	*P* of HWE
Howell	2001	Melanoma	UK	European	HB	165/158	0.69
MartíNez-Escribano	2002	Melanoma	Spain	European	HB	42/48	0.58
Roh	2002	Cervical Cancer	Korea	Asian	HB	144/179	0.72
El–Omar	2003	Esophageal Cancer	USA	Mixed	PB	161/210	0.43
El–Omar	2003	Gastric Cancer	USA	Mixed	PB	314/210	0.43
Heneghan	2003	Hepatocellular Carcinoma	China	Asian	HB	98/97	0.02
Munro	2003	Hodgkin Lymphoma	UK	European	HB	147/110	0.11
Wu	2003	Gastric Cancer	Taiwan	Asian	HB	220/230	0.23
Basturk	2004	Renal Cell Cancer	Turkey	Mixed	HB	29/50	0.32
Maranda	2004	Diffuse Large B-Cell Lymphoma	France	European	HB	199/112	0.54
Savage	2004	Esophageal Cancer	China	Asian	PB	119/386	0.38
Savage	2004	Gastric Cancer	China	Asian	PB	84/386	0.38
Zhang	2004	Basal Cell Carcinoma	China	European	HB	241/260	0.32
Alonso	2005	Melanoma	Spain	European	HB	98/100	0.21
Alpízar-Alpízar	2005	Gastric Cancer	Costa Rica	Mixed	HB	45/45	0.01
Guzowski	2005	Breast Cancer	USA	Mixed	HB	50/25	0.71
Guzowski	2005	Leukemia	USA	Mixed	HB	17/25	0.71
Langsenlehner	2005	Breast Cancer	Australia	European	PB	500/496	0.82
Lee	2005	Gastric Cancer	Korea	Asian	HB	122/120	0.06
Macarthur	2005	Colorectal Cancer	Scotland	European	PB	258/403	0.46
Mazur	2005	Myeloma	Poland	European	HB	54/50	0.22
Shih	2005	Non-small Cell Lung Cancer	Taiwan	Asian	HB	154/205	0.91
Tseng	2005	Hepatocellular Carcinoma	Taiwan	Asian	HB	208/184	0.57
Zambon	2005	Gastric Cancer	Italy	European	HB	129/644	0.70
Zoodsma	2005	Cervical Cancer	Netherlands	European	PB	654/606	0.21
Braicu	2006	Ovarian Cancer	Germany	European	HB	147/129	0.90
Crivello	2006	Colorectal Cancer	Italy	European	HB	62/124	0.72
Kamangar	2006	Gastric Cancer	Filand	European	PB	112/208	0.78
Lan	2006	Non-Hodgkin’s Lymphoma	USA	European	PB	482/563	0.03
Pratesi	2006	Nasopharyngeal Carcinoma	Italy	European	PB	89/130	0.27
Purdue	2006	Non-Hodgkin’s Lymphoma	Australia	European	PB	540/489	0.87
Scola	2006	Breast Cancer	Italy	European	HB	84/106	0.07
Sicinschi	2006	Gastric Cancer	Mexico	European	HB	181/369	0.38
Sugimoto	2006	Gastric Cancer	Japan	Asian	HB	105/168	0.42
Cozar	2007	Colon Cancer	Spain	European	HB	95/175	0.39
Cozar	2007	Renal Cell Cancer	Spain	European	HB	127/175	0.39
Eder	2007	Prostate Cancer	Australia	European	PB	547/545	0.44
Garcia-Gonzalez	2007	Gastric Cancer	Spain	European	HB	404/404	0.08
Gonullu	2007	Breast Cancer	Turkey	Mixed	HB	38/24	0.59
Ivansson	2007	Cervical Cancer	Sweden	European	HB	1282/288	0.33
Purdue	2007	Testicular Germ Cell Tumors	USA	European	PB	505/604	0.28
Vogel	2007	Basal Cell Carcinoma	Denmark	European	PB	304/315	0.92
Wei	2007	Nasopharyngeal Carcinoma	China	Asian	HB	198/210	0.84
Cacev	2008	Colon Cancer	Croatia	European	PB	160/160	0.40
Colakogullari	2008	Lung Cancer	Turkey	Mixed	HB	44/59	0.74
Crusius	2008	Gastric Cancer	Netherlands	European	PB	237/1122	0.05
Erdogan	2008	Thyroid Cancer	Turkey	Mixed	HB	42/113	0.81
Faupel-Badger	2008	Prostate Cancer	Filand	European	PB	511/386	0.54
Kube	2008	Non-Hodgkin’s Lymphoma	Germany	European	HB	500/236	0.35
Vogel	2008	Lung Cancer	Denmark	European	PB	403/744	0.34
Yao	2008	Oral Cancer	China	Asian	HB	280/300	0.81
Zabaleta	2008	Prostate Cancer	USA	African	HB	67/128	0.19
Zabaleta	2008	Prostate Cancer	USA	European	HB	479/401	0.44
Ando	2009	Gastric Cancer	Japan	Asian	HB	330/190	0.06
Kang	2009	Gastric Cancer	Korea	Asian	HB	333/332	0.59
Schoof	2009	Melanoma	Germany	European	HB	164/162	1.00
Tsilidis	2009	Colorectal Cancer	USA	Mixed	PB	203/361	0.58
Wang	2009	Prostate Cancer	USA	Mixed	PB	255/255	0.64
Hart	2010	Non-small Cell Lung Cancer	Norway	European	PB	434/433	0.01
Kong	2010	Breast Cancer	China	Asian	HB	315/322	0.01
Liu	2010	Prostate Cancer	China	Asian	HB	262/270	0.48
Vancleave	2010	Prostate Cancer	USA	African	HB	189/651	0.06
Li	2011	Hepatocellular Carcinoma	China	Asian	PB	150/347	−
Liu	2011	Gastric Cancer	China	Asian	HB	234/243	0.77
Shekari	2011	Cervical Cancer	India	European	HB	200/200	0.05
Zeng	2011	Gastric Cancer	China	Asian	HB	151/153	0.15
Andersen	2012	Colorectal Cancer	Denmark	European	PB	378/775	0.33
He	2012	Gastric Cancer	China	Asian	HB	196/248	0.10
Pooja	2012	Breast Cancer	India	European	HB	200/200	0.08
Zhang	2012	Non-Hodgkin’s Lymphoma	China	Asian	PB	514/557	0.87

HB, hospital based; HWE: Hardy–Weinberg Equilibrium; PB, population based.

### Quantitative Synthesis

**Figure 2:**
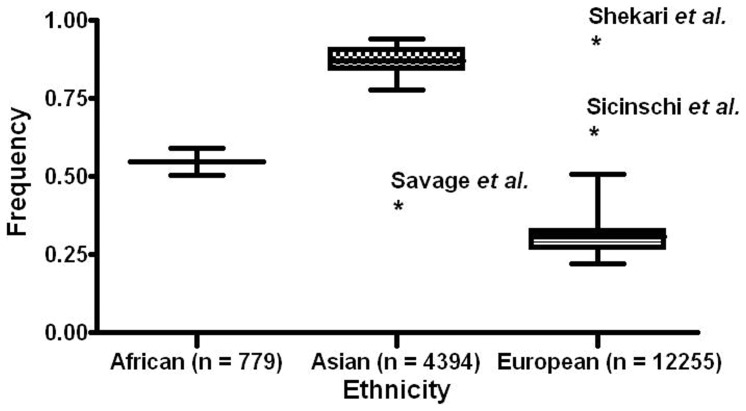
Frequencies of the variant alleles among controls stratified by ethnicity. Asterisks represent outliers.

There was a wide variation in the A allele frequency of the polymorphism among the controls across different ethnicities. For Asian populations, the *IL-10*-592C>A A allele frequency was 0.87 (95% CI = 0.85–0.90), which was significantly higher than that in European populations (0.31, 95% CI = 0.29–0.32, *P*<0.001) (Fig. 2). Overall, individuals with AA genotype had a 0.90 fold lower cancer risk compared with the CC genotype (OR = 0.90, 95% CI = 0.83–0.98, *I* ^2^ = 18.00%). In addition, significant main effects was also observed in a recessive model (OR = 0.92, 95% CI = 0.86–0.98, *I* ^2^ = 25.80%). In a stratified analysis by specific cancer type, we also found decreased risk among studies of smoking-related cancer (OR = 0.77, 95% CI = 0.62–0.96 for AA versus CC, *I* ^2^ = 15.80% for heterogeneity; OR = 0.87, 95% CI = 0.76–0.99 for CA/AA versus CC, *I^ 2^* = 0.00% for heterogeneity). According to ethnicity, significantly decreased risks were also found among the Asian population (OR = 0.79, 95% CI = 0.69–0.91 for AA versus CC, *I* ^2^ = 13.30% for heterogeneity; OR = 0.85, 95% CI = 0.75–0.97 for CA/AA versus CC, *I* ^2^ = 0.00% for heterogeneity; OR = 0.87, 95% CI = 0.80–0.95 for AA versus CC/CA, *I* ^2^ = 34.40% for heterogeneity). In the stratified analysis by source of control groups, we found that the variant genotypes were associated with a significantly decreased risk in hospital-based controls in all genetic model (OR = 0.92, 95% CI = 0.85–0.99 for CA versus CC, *I* ^2^ = 0.00% for heterogeneity; OR = 0.86, 95% CI = 0.77–0.96 for AA versus CC, *I* ^2^ = 9.30% for heterogeneity; OR = 0.91, 95% CI = 0.85–0.98 for CA/AA versus CC, *I* ^2^ = 0.00% for heterogeneity; OR = 0.91, 95% CI = 0.84–0.98 for AA versus CC/CA, *I* ^2^ = 26.60% for heterogeneity). However, no significant associations were found for European and African populations. According to source of controls, no significant associations were observed in population-based studies (Table 2).

**Table 2:** Stratified analyses of the *IL-10* -592C>A polymorphism on cancer risk.

Variables	*n* a	Cases/Controls	CA versus CC		AA versus CC		CA/AA versus CC(dominant)		AA versus CC/CA(recessive)	
			OR (95% CI)	*I* ^2^ (%)	OR (95% CI)	*I* ^2^ (%)	OR (95% CI)	*I* ^2^ (%)	OR (95% CI)	*I* ^2^ (%)
Total	70	16785/19713	0.98 (0.93–1.03)	0.00	**0.90 (0.83–0.98)**	18.00	0.97 (0.92–1.01)	0.80	**0.92 (0.86–0.98)**	25.80
Cancer types										
Breast cancer	6	1187/1173	0.94 (0.78–1.12)	0.00	0.77 (0.59–1.02)	0.00	0.90 (0.76–1.07)	0.00	0.83 (0.66–1.05)	8.60
Cervical cancer	4	2280/1273	1.12 (0.95–1.33)	39.40	1.22 (0.88–1.68)	0.00	1.15 (0.97–1.35)	19.60	1.19 (0.94–1.50)	0.00
Colorectal cancer	6	1156/1998	1.07 (0.92–1.25)	19.40	0.97 (0.56–1.68)^b^	50.50	1.06 (0.92–1.23)	27.50	0.93 (0.54–1.60)^b^	50.30
Gastric cancer	16	3197/5072	0.95 (0.84–1.07)	0.00	0.91 (0.77–1.08)	42.90	0.94 (0.83–1.05)	4.20	0.91 (0.76–1.10)^b^	56.60
Lung cancer	4	1035/1441	0.94 (0.78–1.12)	44.40	0.69 (0.34–1.38)^b^	64.60	0.91 (0.77–1.09)	28.20	0.74 (0.39–1.39)^b^	69.60
Hepatocellularcarcinoma	3	456/628	0.88 (0.52–1.50)	44.50	0.76 (0.45–1.27)	0.00	0.85 (0.57–1.24)	0.00	0.83 (0.60–1.15)	0.00
Melanoma	4	469/468	1.09 (0.83–1.43)	0.00	1.18 (0.71–1.98)	44.70	1.11 (0.86–1.43)	0.00	1.14 (0.69–1.88)	48.60
Non-HodgkinLymphoma	5	2235/1957	0.95 (0.82–1.10)	0.00	0.79 (0.61–1.00)	0.00	0.92 (0.80–1.06)	0.00	0.83 (0.69–1.00)	0.00
Prostate cancer	7	2310/2636	1.01 (0.89–1.15)	36.60	1.04 (0.84–1.30)	0.00	1.03 (0.91–1.16)	43.20	1.02 (0.85–1.24)	0.00
Other cancer	15	2460/3067	0.89 (0.79–1.01)	0.00	0.85 (0.69–1.04)	0.00	0.89 (0.80–1.00)	0.00	0.93 (0.78–1.11)	0.00
Smoking-relatedcancer	11	2038/2902	0.88 (0.77–1.00)	6.70	**0.77 (0.62–0.96)**	15.80	**0.87 (0.76–0.99)**	0.00	0.85 (0.71–1.00)	24.20
Ethnicity										
African	2	256/779	0.98 (0.72–1.34)	0.00	0.88 (0.58–1.35)	0.00	0.96 (0.71–1.28)	0.00	0.89 (0.60–1.32)	0.00
Asian	20	4217/5127	0.90 (0.78–1.03)	0.00	**0.79 (0.69–0.91)**	13.30	**0.85 (0.75–0.97)**	0.00	**0.87 (0.80–0.95)**	34.40
European	37	11114/12430	0.99 (0.94–1.05)	12.30	0.96 (0.86–1.08)	26.50	0.99 (0.94–1.05)	12.20	0.98 (0.88–1.09)	30.00
Mixed	11	1198/1377	0.97 (0.82–1.15)	0.00	1.04 (0.75–1.45)	0.00	0.98 (0.84–1.16)	0.00	1.05 (0.76–1.45)	0.00
Source of controls										
Hospital based	46	8871/9022	**0.92 (0.85–0.99)**	0.00	**0.86 (0.77–0.96)**	9.30	**0.91 (0.85–0.98)**	0.00	**0.91 (0.84–0.98)**	26.60
Population based	24	7914/10691	1.02 (0.96–1.09)	19.20	0.96 (0.84–1.09)	31.30	1.01 (0.95–1.08)	27.20	0.94 (0.84–1.06)	27.00

a Number of comparisons.

b Random-effects estimate.

Smoking-related cancers: lung, esophageal, nasopharyngeal, oral and renal cell cancers.

Bold values indicate significant difference.

### Test for Heterogeneity

In the sub-analysis of gastric cancer, there was significant heterogeneity for recessive model comparison (AA versus CC/CA: *I* ^2^ = 56.60% for heterogeneity). Then, we assessed the source of heterogeneity for recessive model comparison (AA versus CC/CA) by ethnicity and source of controls. As a result, neither ethnicity (χ^2^ = 5.06, df = 2, *P* = 0.08) nor source of controls (χ^2^ = 0.01, df = 1, *P = *0.91) was found to contribute to substantial heterogeneity.

### Sensitivity Analyses

Sensitivity analyses indicated that two independent studies by Wu *et al.* [50] was the main origin of heterogeneity in the sub-analysis of gastric cancer. The heterogeneity was effectively decreased or removed by exclusion of the study (AA versus CC/CA: *I* ^2^ = 46.90%). Although the genotype distribution in five studies did not follow Hardy-Weinberg equilibrium, the corresponding pooled ORs were not materially altered by including the studies. In addition, no other single study influenced the pooled OR qualitatively, as indicated by sensitivity analyses, suggesting that the results of this meta-analysis are stable.

### Publication Bias

**Figure 3:**
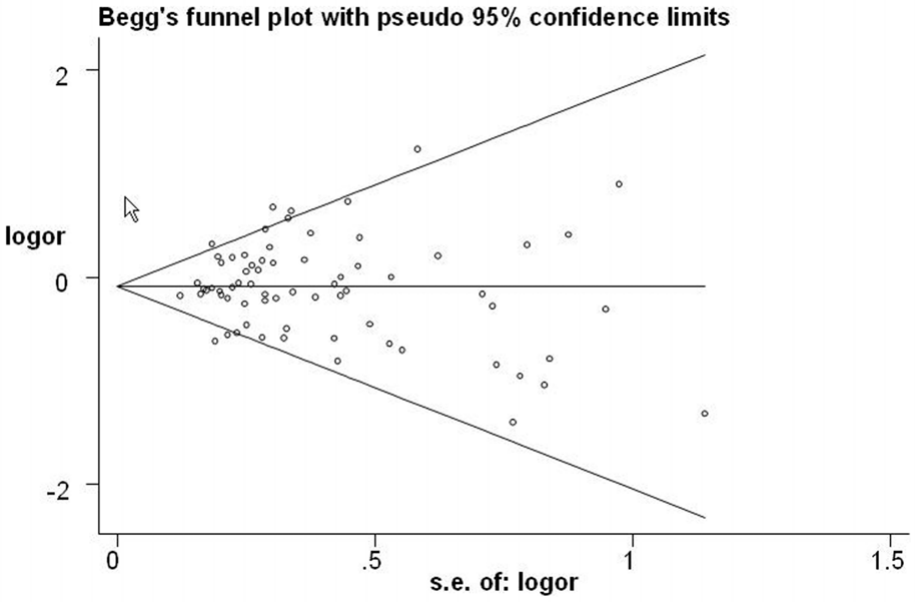
Begg’s funnel plot for publication bias test (AA vs. CC/CA). Each point represents a separate study for the indicated association. Log [OR], natural logarithm of odds ratio. Horizontal line, mean effect size.

Begg’s funnel plot and Egger’s test were performed to assess the publication bias of literatures. As shown in Fig. 3, the shapes of the funnel plots did not reveal any evidence of obvious asymmetry in all comparison models. Thus, Egger’s test was used to provide statistical evidence of funnel plot symmetry. The results still did not show any evidence of publication bias (*t* = −1.38, *P* = 0.172 for CA versus CC; *t* = −0.86, *P* = 0.390 for AA versus CC; *t* = −1.99, *P* = 0.051 for AA/CA versus CC; *t* = −0.12, *P* = 0.903 for AA versus CC/CA).

## Discussion

The present meta-analysis investigated the association between the *IL-10-592C>A* polymorphisms and cancer risk, based on 70 published case–control studies from 65 articles. The results provided evidence that the *IL-10*-592C>A polymorphism was associated with a significant decrease in overall cancer risk. The variant homozygote genotype AA of the *IL-10*-592C>A polymorphism, was associated with a modest but significantly decreased risk in homozygote comparison and recessive model. In the stratified analysis, the risk remained for studies of smoking-related cancer, Asian populations and hospital-based studies. Given the important roles of IL-10 in carcinogenesis, it is biological plausible that *IL-10* polymorphism might modulate the risk of cancer.

A common [ATA] haplotype is formed by polymorphisms at positions -1082,-819 and -592 in the promoter of *IL-10* gene [83], [84]. It has been reported that the -592 A variant, the -1082 A variant as well as the [ATA] haplotype was associated with lower IL-10 expression [51], [85], [86]. Thus, the -592 A variant can be regarded as a low-producer allele of the *IL-10* gene. Research has showed that increased serum IL-10 levels could facilitate development of tumors by suppressing the expression of MHC class I and II antigens [87] and preventing tumor antigen presentation to CD8-cytotic T lymphocytes. It has been revealed that the homozygous *IL-10-592AA* genotype, indicating homozygosity for the [ATA] haplotype, was protective against breast cancer [51]. Similarly, elevated serum levels of IL-10 were found in non-small cell lung cancer patients; moreover, IL-10 serum levels were shown to be higher in patients with metastatic disease when compared to those with undisseminated cancer [88]. Consistently, we also found that individuals with the AA genotype which exhibits low production of IL-10 were associated with a lower cancer risk than participants with the CC genotype in our meta-analysis.

Tobacco smoking is a well-established risk factor for cancers of many organs, including lung, esophagus, oral cavity, pharyngeal and kidney [89]–[92]. Tobacco use has been proven to affect the immune system and influence the production of IL-10 [93]. Also, studies showed that smokers have impaired T lymphocyte suppressor cell function and decreased natural killer cell activity compared with non-smokers. Furthermore, IL10 might protect tumors by inhibiting cytotoxic T lymphocyte (CTL)-mediated tumor-specific cell lysis [87], [94]. Further studies are needed to investigate the relationship.

Our results indicated that the AA variant genotype was associated with decreased risk in smoking-related cancer but not for breast cancer, cervical cancer, colorectal cancer, esophageal cancer, gastric cancer, melanoma, lung cancer, hepatocellular carcinoma, Non-Hodgkin lymphoma or prostate cancer. One possible explanation for the discrepancy is that carcinogenic mechanism underlying the etiology may differ by different tumor sites and that the *IL-10* genetic variants may play a different role in different cancers. Even at the same tumor site, considering the relatively small sample size in some studies and the possible small effect size of this genetic polymorphism to cancer, the discrepancy will become apparent since some of these studies may have insufficient statistical power to detect a small but real association. For example, there were only three studies included in the analysis with limited sample size for esophageal cancer, with 456 cases and 628 controls, so the results may be capricious and should be interpreted with caution.

In the subgroup analysis by ethnicity, we found an evidence for the association between the *IL-10*-592C>A polymorphism and cancer risk among Asians but not among Europeans or Africans, suggesting a possible role of ethnic differences in genetic backgrounds and the environment they lived in (Table 2). Several concerns may account for it. First, nine of the 20 Asian studies investigated gastric cancer (weighted 36.68% and 42.24% in comparison of AA versus CC and AA versus CC/CA), whereas only five out of the 37 studies focused on gastric cancer in the European population (weighted 18.02% and 18.48% in comparison of AA versus CC and AA versus CC/CA). Second, the prevalence of variant allele of *IL-10*-592 C>A polymorphism among the controls varies markedly with ethnicity. For Asian populations, the *IL-10*-592C>A A allele frequency was 0.87 (95% CI = 0.85–0.90), which was significantly higher than that in European populations (0.31, 95% CI = 0.29–0.32, *P*<0.001). Other factors such as different matching criteria, selection bias and adjustment in the statistical analyses, misclassification on disease status and genotyping method might also play a role. In addition, there are only two reported studies and limited number of patients was available for African, which limited us to detect stable effects in this population. Additional studies are warranted to further validate ethnic difference in the effect of the *IL-10*-592C>A polymorphism on cancer risk, especially in Africans.

When stratifying the source of controls, a moderate strength was observed in hospital-based controls, but not the population-based controls. The discrepancy may result from a differential effect of selection criteria in different cancers, as well as the weight of each study, which was dictated by sample size in the meta-analysis. Another reason may be that the hospital-based studies have some inherent selection biases as such controls may just represent a sample of ill-defined reference population and may not be very representative of the study population or the general population, particularly when the genotypes under investigation were associated with the disease conditions that the hospital-based controls may have.

One of the most important goals of the meta-analysis is to identify the source of heterogeneity. In the sub-analysis of gastric cancer, there was significant heterogeneity for recessive model comparison. Thus, we stratified the studies on gastric cancer according to ethnicity and source of controls. Through analysis, it was found that neither ethnicity nor source of controls contribute to substantial heterogeneity. It is possible that other unmeasured characteristics in different study populations and/or inherited limitations of the recruited studies may partially contribute to the observed heterogeneity.

The meta-analysis has some strengths and limitations. This article is potentially limited in several ways. First, our result was based on unadjusted estimates, while a more precise analysis should be conducted adjusted by other factors such as age, sex, smoking and drinking status. Lack of information for data analysis might cause serious confounding bias. In addition, lacking the original data of the included studies limited our further evaluation of the potential interactions because gene–gene, gene–environment interactions and even different polymorphic loci of the same gene might modulate cancer risk. Second, some of the studies had a very small sample size and did not have adequate power to detect the possible risk for *IL-10*-592C>A polymorphism and the observed significant ORs in some studies of small sample size may be false association. Third, misclassifications on genotypes and disease status may influence the results because the quality control of genotyping was not well documented in some studies and cases in several studies were also not confirmed by pathology or other gold standard methods. Nonetheless, advantages in our meta-analysis should also be acknowledged. First, the statistical power of the analysis was greatly increased, because a substantial number of cases and controls were pooled from different studies. Second, the quality of case–control studies included in this meta-analysis was satisfactory according to our selection criteria. Third, we did not detect any publication bias indicating that the whole pooled result should be unbiased.

In conclusion, our meta-analysis suggests that the *IL-10*-592C>A polymorphism was associated with a significant decrease in overall cancer risk, especially in smoking-related cancer, Asians and hospital-based studies. However, large studies using standardized unbiased genotyping methods, enrolling precisely defined cancer patients and well-matched controls, especially in African populations, with more detailed individual and environmental data are warranted to validate the results of our meta-analysis.
